# Supplementary material for: miR-383-5p Regulates Preadipocyte Proliferation and Differentiation by Targeting RAD51AP1
Source: Int J Mol Sci. 2023 Sep 13;24(18):14025. doi: 10.3390/ijms241814025 (PMC10531573; doi:10.3390/ijms241814025)
Supplement: Supplementary file 1 [file ijms-24-14025-s001.zip › Supplementary/Table S9.docx]

**Table S9.** The sequence information of RNA oligo.

| Name | Sequence Information (5’-3’) |
| --- | --- |
| miR-383-5p mimic | F: AGAUCAGAAGGUGAUUGUGGCU |
|  | R: CCACAAUCACCUUCUGAUCUUU |
| miR-383-5p NC | F: UUGUACUACACAAAAGUACUG |
|  | R: GUACUUUUGUGUAGUACAAUU |
| miR-383-5p inhibitor | AGCCACAAUCACCUUCUGAUCU |
| miR-383-5p INC | CAGUACUUUUGUGUAGUACAA |
