# Supplementary material for: miR-383-5p Regulates Preadipocyte Proliferation and Differentiation by Targeting RAD51AP1
Source: Int J Mol Sci. 2023 Sep 13;24(18):14025. doi: 10.3390/ijms241814025 (PMC10531573; doi:10.3390/ijms241814025)
Supplement: Supplementary file 1 [file ijms-24-14025-s001.zip › Supplementary/Table S8.docx]

**Table S8.** Primer information for each gene.

| Gene Symbol | Primer Sequences (5’-3’) |
| --- | --- |
| IGF2BP2 | F: TGCCTCGTGACCAAACACCA |
|  | R: GGTGCATAGGTACAGGGAACTGG |
| PRR16 | F: ACCTGTGAAGTGCGAGGAGC |
|  | R: GGCAAGCCTCCGTTTCTGAG |
| BCL9 | F: GCATGCCTCCGCAGATAGG |
|  | R: ACATGGGAAGGAACGGCCTG |
| PPARγ | F: CCTTGCTGTGGGGATGTCTC |
|  | R: ATGCGGATGGCGACTTCTTT |
| C/EBPα | F: AGGAGTAACCGTGTGCCTTG |
|  | R: CACCTCACCTCATTGGTCCC |
| FABP4 | F: TGAAAGAAGTGGGAGTGGGC |
|  | R: CACCACCAGTTTATCGCCCT |
| GAPDH | F: CTTCGGCATTGTGGAGGG |
|  | R: GGAGGCAGGGATGATGTTCT |
| RAD51AP1 | F: CGACACTGCTGATGACACTGACC |
|  | R: TCTTTTCTACTGGGGATTTTACCTTC |
| ACC | F: GTGGTCTTCGTGTGAACTGG |
|  | R: TTCTTCTGCTGCCTTTAGCC |
| FAS | F: ACCACGTCCAAGGAGAGCA |
|  | R: AGTTCTGCACCGAGTTGAGC |
| SCD | F: CTTGCGATATGCTGTGGTGC |
|  | R: AAGTTGATGTGCCAGCGGTA |
| CN1H4 | F: GCACTGGTTCATCTTCCTTCTC |
|  | R: ATGTGTGACTTCAGTTGCCCTC |
| NUF2 | F: GAGGTGCTGTCTGTGAGCGAGT |
|  | R: CGGTGGCATCTTTTAGTTGTTGA |
| U6 | F: GGAACGATACAGAGAAGATTAGC |
|  | R: TGGAACGCTTCACGAATTTGCG |
| miR-383-5p | CCGGCAGATCAGAAGGTGATT |
