# Supplementary material for: miR-383-5p Regulates Preadipocyte Proliferation and Differentiation by Targeting RAD51AP1
Source: Int J Mol Sci. 2023 Sep 13;24(18):14025. doi: 10.3390/ijms241814025 (PMC10531573; doi:10.3390/ijms241814025)
Supplement: Supplementary file 1 [file ijms-24-14025-s001.zip › Supplementary/Table S1.docx]

**Table S1.** Mimic and NC groups data output quality statistics.

| Sample | Clean Reads | Q20 (%) | GC (%) | Total Mapping (%) |
| --- | --- | --- | --- | --- |
| Mimic 1 | 45891138 | 97.40 | 52.88 | 89.71 |
| Mimic 2 | 40029696 | 97.50 | 53.36 | 89.60 |
| Mimic 3 | 45606056 | 97.42 | 53.60 | 89.05 |
| NC 1 | 44971560 | 97.62 | 52.73 | 90.23 |
| NC 2 | 43716954 | 97.39 | 53.36 | 89.17 |
| NC 3 | 42292752 | 97.18 | 53.91 | 88.56 |
