# Supplementary material for: miR-383-5p Regulates Preadipocyte Proliferation and Differentiation by Targeting RAD51AP1
Source: Int J Mol Sci. 2023 Sep 13;24(18):14025. doi: 10.3390/ijms241814025 (PMC10531573; doi:10.3390/ijms241814025)
Supplement: Supplementary file 1 [file ijms-24-14025-s001.zip › Supplementary/Table S7.docx]

**Fig. 6.** miR-383-5p regulates the proliferation and differentiation of rabbit preadipocytes by targeting *RAD51AP1*.

1、β-actin：


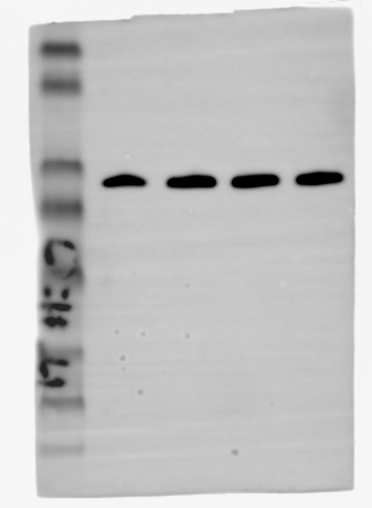


NC Mimic INC Inhibitor

2、SREBP1：


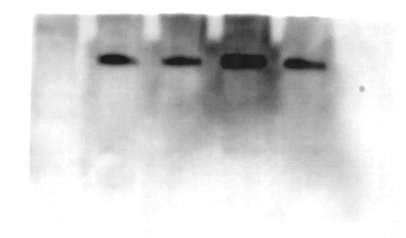


NC Mimic INC Inhibitor

2、FABP4：


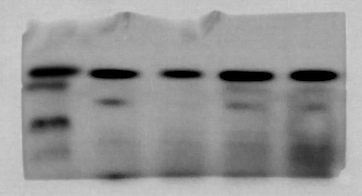


NC Mimic INC Inhibitor
